# The First Investigation of Tick Vectors and Tick-Borne Diseases in Extensively Managed Cattle in Alle District, Southwestern Ethiopia

**DOI:** 10.1155/2020/8862289

**Published:** 2020-12-19

**Authors:** Asrat Solomon, Bereket Molla Tanga

**Affiliations:** ^1^Faculty of Veterianry Medicine, Hawassa University, P.O. Box: 05, Hawassa, Ethiopia; ^2^Alle District Livestock and Fisheries Development, Alle, Ethiopia; ^3^College of Veterianry Medicine, Chungnam National University, Daejeon, Republic of Korea

## Abstract

A cross-sectional study was conducted from March 2019 to February 2020 with the objective of identifying ixodid ticks and haemoparasites, in extensively managed livestock, in Alle district, Southwestern Ethiopia. The study area is assumed to be free from ticks, and there had been no diagnostic and treatment options for tick-borne diseases. Among 384 heads of cattle examined for tick infestation and haemoparasites, 139 (36.19%) were infested with one or more tick species and 25 (6.51%) were haemoparasitised. Two genera of ticks, *Amblyomma* and *Rhipicephalus* formerly (*Boophilus*), and four species (*Amblyomma variegatum, Amblyomma lepidum, Rhipicephalus microplus,* and *Rhipicephalus annulatus*) were identified. The haemoparasite identified was *Babesia bovis*. Among the risk factors, body condition score and season of the year were found to be significantly associated with tick infestation with *x*^2^ = 9.919, *p* > 0.05 and *x*^2^ = 6.216, *p* > 0.05, respectively, at 95% CI. Tick infestation was found to be significantly associated with haemoparasitemia with *x*^2^ = 22.2 and *p* > 0.05, at 95% CI. The finding of the current study is an alarm ring, as the veterinary service had been not considering any haemoparasitemia in the potential list of differential diagnosis and no treatment inputs have been availed for that purpose. Thus, it is recommended that the veterinary service delivery system in the area should take haemoparasites diagnosis and avail treatment alternatives, particularly tick-borne diseases. Furthermore, there should be a strategical approach in controlling tick-borne diseases in the area before the tick-borne diseases get prevalent and where the control after high prevalence could not be easy in extensive livestock management.

## 1. Introduction

Ethiopia possess huge number of livestock populations with an estimated 57.83 million cattle, 29.33 millions of sheep, 29.11 millions of goats, 1.16 million of camels, 9.86 millions of equines, and 56.87 millions of chickens which represent an immense economic potential [1]. Despite this huge resource, it contributes limited share of household and national economy in the country [2].

Ticks are small, wingless ectoparasitic arachnid arthropods that are cosmopolitan and prevalent in warmer climate [3]. Ixodid ticks are one of the most common and harmful blood sucking ectoparasites of cattle worldwide. They are responsible for a wide range of livestock health problems in several countries of the world. They reduce the cattle productivity, milk yield, and skin and hide quality and increase susceptibility to other diseases [4].

Ticks are harmful blood sucking external parasites of livestock that are distributed in all agroecological zones in Ethiopia [5]. Hard ticks (Acari, *Ixodidae*) are ubiquitous blood feeding ectoparasites that infest human and animals and are vectors of pathogenic microorganisms of rickettsial, bacterial, viral, and protozoal origin that cause severe infectious diseases in humans and livestock [6].

Tick-borne pathogens affect 80% of the world's cattle population and are widely distributed throughout the world, particularly in the tropics and subtropics [7]. According to [8], ticks which are considered to be the most important to the health of domestic animal in Africa comprise about seven genera. Among these genera, the main tick genera found in Ethiopia includes *Amblyomma*, subgenus *Rhipicephalus* (*Boophilus*), *Haemaphysalis, Hyalomma*, and *Rhipicephalus*. The genus *Amblyomma* and *Rhipicephalus* are predominating in many parts of the country, and *Hyalomma* and subgenus *Rhipicephalus* (*Boophilus*) also have a significant role [9].

In Ethiopia, there are 47 species of ticks found on livestock and most of them have importance as vector and disease-causing agents and also have a damaging effect on the skin and hide production as reviewed by [10]. Among several tick species widely distributed in Ethiopia, the major tick genera reported are *Amblyomma, Rhipicephalus* (*Boophilus*), *Hyalomma*, and *Haemaphysalis* [11]. The country' environmental condition and vegetation are highly conducive for ticks and tick-borne disease maintenance [12]. Acaricide application is still the main method of tick control in Ethiopia [13]. Currently, organophosphates are the most widely used chemicals although evidence of resistance is emerging [14].

Tick-borne diseases are one of the most constraints to livestock production in developing countries. The most important tick-borne diseases in sub-Saharan Africa are theileriosis caused by *Theileria parva*, babesiosis caused by *Babesia bovis* and *Babesia bigemina*, anaplasmosis caused by *Anaplasma marginale*, and heart-water caused by *Ehrlichia ruminantium* [15].

*Rhipicephalus* is the principal vector for *Babesia bovis* and *Babesia bigemina*. The vectors are widely spread in tropical and subtropical countries [16]. Tropical theileriosis also known as Mediterranean Coast Fever is extremely fatal and debilitating tick-transmitted disease infecting cattle [17]. The two most pathogenic and economically important Theileria species are *Theileria parva* and *Theileria annulata* [18]. Bovine theileriosis is reported in Ethiopia for the first time in the recent study by Gebrekidan et al. [19] in which four species of *Theileria* such as *Theileria velifera*, *Theileria mutans*, *Theileria orientalis* complex, and *Theileria annulata* were identified in cattle in Humera.

Bovine anaplasmosis is one of the most important vector-borne infectious diseases in cattle mainly caused by *Anaplasma marginale* and rarely by *Anaplasma centrale*. The organisms are Gram-negative obligate intracellular rickettsial bacteria [20], classified in the genus *Anaplasma*, belonging to the family Anaplasmataceae of the order Rickettsiales [21]. *Anaplasma phagocytophilum* has been recognized as an animal pathogen and is an emerging human pathogen of public health relevance [22].

There are different routes of haemoparasite transmission; the main route is by ticks which act as vectors for many haemoprotozoal diseases such as *Babesia*, *Theileria*, and *Anaplasma* [23]. Other routes include intrauterine, colostral, and mechanical transmission [24].

Due to economic and veterinary importance of ticks, their control and transmission of tick-borne diseases remain a challenge for the cattle industry of the world, and it is priority for many countries in tropical and subtropical regions [25]. Ticks are effective disease vectors, second only to mosquitoes in transmitting infectious diseases [26]. Haemoparasites have a great economic impact on livestock affecting 80% of the world cattle population and causes economic loss due to morbidity and mortality. They also impair the export and import trade of live animal and animal products by downgrading their quality [27].

Among livestock, cattle play a significant socioeconomic role in the livelihoods of the Ethiopian people [28]. Ticks and tick-borne diseases affect the productivity of cattle and leads to a significant adverse impact on the livelihoods of resource poor farming communities [29].

The importance of strategies to control the diseases requires more detailed knowledge of their prevalence and how they interact with each other [30]. No study has been conducted regarding identification of ixodid ticks and tick-borne haemoparasites in this area. And there were no diagnostic and treatment approaches in place even to detect whether there is a problem or not. Therefore, the current study was aimed to investigate cattle ixodid ticks and tick-borne haemoparasite in Alle district.

## 2. Materials and Methods

### 2.1. Study Area

Alle district located 650 km from Addis Ababa, the capital, with an altitude of 590–2800 meters above sea level and annual rain fall ranges from 800 mm to 1500 mm. The weather condition of the area is arid (35%), semiarid (47%), and humid (18%), which makes the area suitable for production of different crops [31]. The temperature ranges from 20°C to 23°C [31]. The livestock population of Woreda has a total head of 509,551 cattle, 511,107 goats, 78,765 sheep, 18,534 donkeys, 519 horses, 383 mules, and 227,846 chickens [31].

### 2.2. Study Population

The target population was all cattle in the 7 kebeles, the lowest administrative units, in Alle district. The sample population was randomly selected cattle from animals coming to veterinary clinics and working and resting places in Alle district.

### 2.3. Sampling Method and Sample Size Determination

The sample size was determined according to the random sampling approach. 5% of desired absolute precision at the confidence level of 95% was used. Since no study was conducted in this area regarding this topic, 50% prevalence was used. The sample size was determined using the equations given by Thrusfield [32].

Whereby,

(1) $$\begin{array}{c} n=\frac{Z^{2}-PQ}{e^{2}}, \end{array}$$

where *Q* = 1 − *P*, *Z* = 1.96, *e* = precision error (0.05), and *P* = expected prevalence of about 50%. Therefore, *n* = 1.96^2^ × (0.5) (1 − 0.5)/(0.05^2^) = 384.

### 2.4. Study Design and Methodology

A cross-sectional study was conducted on cattle to determine the prevalence of ixodid ticks and tick-borne haemoparasites in cattle in Alle district from March 2019 to February 2020.

#### 2.4.1. Collection and Identification of Ticks

The entire body surface of the cattle was inspected for the presence of ticks. After fully restraining the animals, all visible adult tick species were removed by hands and using forceps holding the basis capitulum, so as not to lose the mouth parts of the ticks. Ticks from each animal were collected and placed in separate prelabeled universal bottles containing 10% formalin solution until identification.Date of collection, age of animal, sex of animal, body condition score (BCS), and season of the year were recorded as well. The age of animals was grouped as young (between 1 and 3 years) and adults (>3 years) according to the classification method used by [33], while body condition scores of animals were evaluated during sample collection. They were classified as emaciated (poor), moderate (medium), and good based on anatomical parts and the flesh and fat cover at different body parts [34]. Extremely lean, having prominent dorsal spines pointed to touch and individual visible transverse processes into which a finger could be easily pushed, was considered as a poor body condition score. A medium body condition score cattle was expressed as having visible ribs with little fat cover and barely visible dorsal spines. A good body condition score was given for the animals when fat cover was easily seen in critical areas and felt and the transverse processes were seen or felt. Ticks were counted and subsequently identified to the genus level and species level by using a direct stereomicroscope using key morphological characteristic, i.e., size of mouth parts, color of the body, leg color, presence or absence of the eye, shape of scutum, body, coxae one, festoon, and ventral plates were considered as described by Walker et al. [8].

#### 2.4.2. Collection and Examination of Blood Sample

Blood film examination was performed with Giemsa staining procedures, and microscopic examination of slides was conducted according to [35]. Blood was taken from the ear veins, and thin blood smears were made and labeled. The slide was then air dried and immediately fixed with absolute methyl alcohol for few seconds, and then, the smear was stained with 10% Giemsa stain. Finally, the slides were thoroughly examined under a compound microscope using oil immersion. The parasites searched for include *Babesia species* (spp.), *Anaplasma* spp., and *Theileria* spp.

### 2.5. Data Management and Analysis

The data collected were stored in the Microsoft Excel Spreadsheet and analyzed by using Stata Version 13. Descriptive statistics were used to summarize the data. The prevalence was calculated as the total infestation/infection cases divided by the total cattle examined. Pearson's chi-square statistics were used to test the association between variables. *P* value less than 0.05 at 95% confidence level was considered as significance cut value in interpreting the results.

## 3. Results

In this study, a total of 384 cattle were examined. Two tick genera, *Rhipicephalus* and *Amblyomma*, and four tick species were identified. The tick species identified were *Amblyomma variegatum (A. variegatum), Amblyomma lepidum* (*A. lepidum*), *Rhipicephalus microplus* (*R. microplus*), and *Rhipicephalus annulatus* (*R. annulatus*) in order of relative infestation rates as shown in Table 1. The breeds of animals examined were 380 (98.95%) local, 1 exotic (0.26%), and 3 (0.78%) crossbreed's.

### 3.1. Prevalence of Ixodid Ticks

Among 384 cattle examined for tick infestation, 139 (36.19%) were infested with ticks. The tick genera identified are illustrated in Table 2. Prevalence of ticks varied in different kebeles of the study area. Highest prevalence was observed in Guma kebele (57.44%), followed by Gawada (41.9%), and lowest prevalence was observed at Addis Oltima kebele (19.04%) (Table 3).

### 3.2. Risk Factors Associated with Ixodid Tick Infestation

Age, sex, and breed were not significantly associated with tick infestation, whereas the body condition score and season of the year were found statistically significantly associated with tick infestation in cattle. The prevalence of tick infestation was higher in older, female, and exotic breed of animals, though it is not statistically significant. Regarding the body condition score and season, tick infestation was higher in poor body condition animal and in wet season of the year (Table 4).

### 3.3. The Prevalence of Haemoparasites

Among 384 cattle examined for tick-transmitted haemoparasites parasitism, 25 (6.51%) were found parasitized. Among the 25 animals having haemoparasites, majority, 20 (80%) were infested with ticks during the study period. The haemoparasite identified is *Babesia bovis* in all animals tested positive (Table 5).

### 3.4. Risk Factors Associated with the Prevalence of Haemoparasites

Age, sex, body condition score, breed, and season were not statistically significantly associated with haemoparasitism. Only tick infestation of animals was found statistically significantly associated with haemoparasitism. Higher prevalence of haemoparasitism was observed in old, female, medium body conditioned, and local breed animals, eventhough it is not statistically significantly different. Regarding seasonal variation of tick infestation, the prevalence was found higher in wet season than dry season of the year (Table 5).

## 4. Discussion

The current study identified the overall prevalence of 36.19% of tick infestation in cattle in Alle district, which is lower than the report by Meseret et al. [36] who reported 59.6% prevalence in Harari region, Eastern Ethiopia. This might be due to the difference in area coverage of the study area and agroecology. It might also be due to a variation in acaricide application and access to acaricide as by Nath et al. [37] and the method of application of acaricides [38], as chemical acaricide is the main weapon for the control of ticks.

Tick infestation was reported in different parts of Ethiopia and is higher as compared to the finding of the current study, the finding of Mesfin et al. [39] (89.1%) in Wolaita Zone, the finding of Kumisa et al. [40] (68.8%) in Dandi district, Oromia, the finding of Kemal et al. [41] (75.7%) in Arbegona district, and the finding of Wasihun and Doda [42] (61%) in Humbo district. The difference in prevalence could be attributed to the difference in environmental factors such as humidity which are conducive for survival and growth of tick developmental stages, and reproduction of ticks varying among study areas. Environmental factors which support tick survival in the specific area include temperature, humidity, rainfall [43], vegetation [44], host availability, and season [45]. The variation in prevalences of tick infestation could be also due to variation in animal husbandry and management among the areas, as management found to affect tick prevalence as described by Soberanes-Céspedes et al. [46].

The finding of the current study is also lower than the report of Kemal and Abera [5] who reported 72.1% in Dassenech district, Southern Ethiopia. This could be due to the variation in host range and diversity of wild life between study areas, where in the later, there were high interaction of domestic and wild animals. Peter et al. [47] signified that wild herbivores support a large number of tick species, and Tonetti et al. [48] also described that wild herbivores are believed to be significant reservoirs of tick-borne pathogens.

Regarding the preference of ticks to different sexes of animals, Yakubu et al. [49], Utech and Wharton [50], and Burrow et al. [51] reported high prevalence of tick infestation in male than female animals which is in agreement with the current finding. In the current study area, male animals move from place to place for searching of feed and in this process get infested with ticks, while female animals confined to home for breeding and milking purpose in the respective study areas could had been contributed to the differences in tick occurence between species.

Previous studies in Egypt [52–54], in Pakistan, and [55] in Ethiopia reported a higher prevalence of ticks in exotic breeds, which is in agreement to the current study. But in the current study, only 1 exotic and 3 crossbreeds were observed, and, it is not a sufficient sample size to compare and make interpretation. Although the mechanism of resistance acquired against tick infestation by indigenous breeds is not fully understood, it could be attributed to preimmunity against ectoparasites, which often established through contacts with the parasites at the early stage of their life [52].

Overall prevalence of haemoparasites was 25 (6.51%), and only *Babesia bovis* species was identified as haemmoparasite. This finding is not in line with the reports of studies by Bariso and Worku [56] who reported 11.4% overall haemoparasitemia in Arsi, Central Ethiopia, Lemma et al. [57] who reported 23% in Jimma, and Alemayehu [58] who reported 12% in Jimma, Western Ethiopia. This variation could be attributed to differences in the study area, the level of animal's exposure to ticks (depending on the type of management), and the land use of the area, which are different amongthe study areas.

The prevalence of babesiosis in the current study is in agreement with the report of Ahmad and Hashim [59] who reported 6.6% prevalence from Pakistan and different researchers from different parts of Ethiopia. Lower prevalence of bovine babesiosis in Ethiopia was reported by Bariso and Worku [56] (1.3%) and Shane et al. [60] (1.6%) in Arsi, Wodajnew et al. [61] (1.24%) in Benishangul Gumuz, Sitotaw et al. [62] (0.3%) in Debrezeit, Ethiopia, and Ola-Fadunsin et al. [63] (1.2%) in Nigeria. The differences in prevalence of babesiosis in cattle could be due to the type of diagnosis followed and the differences in different seasons of the year, as it is affected by the tick infestation state [64].

The finding of current study is an alarm for the local policy makers and administrators of the veterinary services, as ticks were not considered to be prevalent and ignored in the livestock diseases causation. Thus, the authors strongly advise that there should be preparedness in diagnosing tick-borne diseases and avail treatment options for the tick-borne diseases, at least for babesiosis.

## 5. Conclusion and Recommendations

In conclusion, 36.19% overall prevalence of ticks and 6.51% prevalence of babesiosis is high enough to be responsible for loss of production, particularly the skin and hide, as there were no prevention and control in place. To this end, tick control in Alle district should be considered, and appropriate preparedness should be in place in the veterinary service delivery system for the diagnosis and treatment of tick-borne diseases.

## Figures and Tables

**Table 1:** Prevalence of tick based on species observed.

Species of tick	Frequency (n)	Percentage (%)
*A. variegatum*	76	54.67
*A. lepidum*	13	9.35
*R. microplus*	27	19.4
*R. annulatus*	11	7.9
Mixed	12	8.63
Total	139	36.19

**Table 2:** Prevalence of tick based on the genera observed.

No.	Species of tick	Frequency (n)	Percentage (%)
1	*Amblyomma*	89	23.17
2	*Boophilus*	38	9.895
3	Mixed	12	3.125
	Total	139	36.19

**Table 3:** Prevalence of tick at kebeles observed.

Name of kebele	Number examined	Number ^+ve^	Percentage (%)
Wolango	91	35	38.46
Guma	47	20	57.44
Gawada	62	26	41.9
Kerkerte	62	26	41.9
Kerkerte	87	33	37.9
Goroze	42	9	21.29
Addis Oltima	21	4	19.04
Lokite	34	12	37.5
Total	384	139	36.19

**Table 4:** Characters of the animal and prevalence of tick.

Animal factors		Frequency (n)	Tick infested	Percentage (%)	*x* ^2^	*P* value
Age	Young	314	109	34.7	1.64	0.200
	Old	70	30	42.85		

Sex	Male	155	54	34.83	0.20	0.064
	Female	229	85	37.11		

BCS	Good	280	94	33.57	9.219	0.010
	Medium	39	11	28.2		
	Poor	65	34	52.3		

Breed	Local	380	137	35.67	1.77	0.411
	Exotic	1	1	100		
	Cross	3	1	33.33		

Season	Wet	148	65	43.9	6.216	0.013
	Dry	236	74	31.35		

Total	384	139	36.19			

**Table 5:** Host factors and prevalence of haemoparasites.

Animal factor		Frequency (n)	Infected	Percentage (%)	*x* ^2^	*P* value
Age	Young	314	17	5.41	3.402	0.065
	Old	70	8	11.4		

Sex	Male	155	11	7.096	0.146	0.702
	Female	229	14	6.11		

BCS	Good	280	18	6.43	3.81	0.149
	Medium	39	5	12.8		
	Poor	65	2	3.07		

Breed	Local	380	25	6.51	0.728	0.869
	Exotic	1	0	0		
	Cross	3	0	0		

Tick infestation	Yes	139	20	14.38	22.2	0.000
	No	245	5	2.04		

Season	Wet	148	14	9.46	3.44	0.064
	Dry	236	11	4.66		

Total	384	25	6.51			

## Data Availability

The data used to support the findings of this study are available at Hawassa University and Alle District Livestock and Fisheries Resources Development Bureau.
